# Supplementary material for: TracrRNA reprogramming enables direct PAM-independent detection of RNA with diverse DNA-targeting Cas12 nucleases
Source: Nat Commun. 2024 Jul 13;15:5909. doi: 10.1038/s41467-024-50243-x (PMC11246509; doi:10.1038/s41467-024-50243-x)
Supplement: Supplementary file 1 — Supplementary Information [file 41467_2024_50243_MOESM1_ESM.pdf]

SUPPLEMENTARY INFORMATION

TracrRNA reprogramming enables direct, PAM-independent detection of RNA with diverse DNA-targeting Cas12 nucleases

Chunlei Jiao, Natalia L. Peeck, Jiaqi Yu, Mohammad Ghaem Maghami, Sarah Kono, Daphne Collias, Sandra L. Martinez Diaz, Rachael Larose and Chase L. Beisel†

†Correspondence: chase.beisel@helmholtz-hiri.de (to C.L.B.)

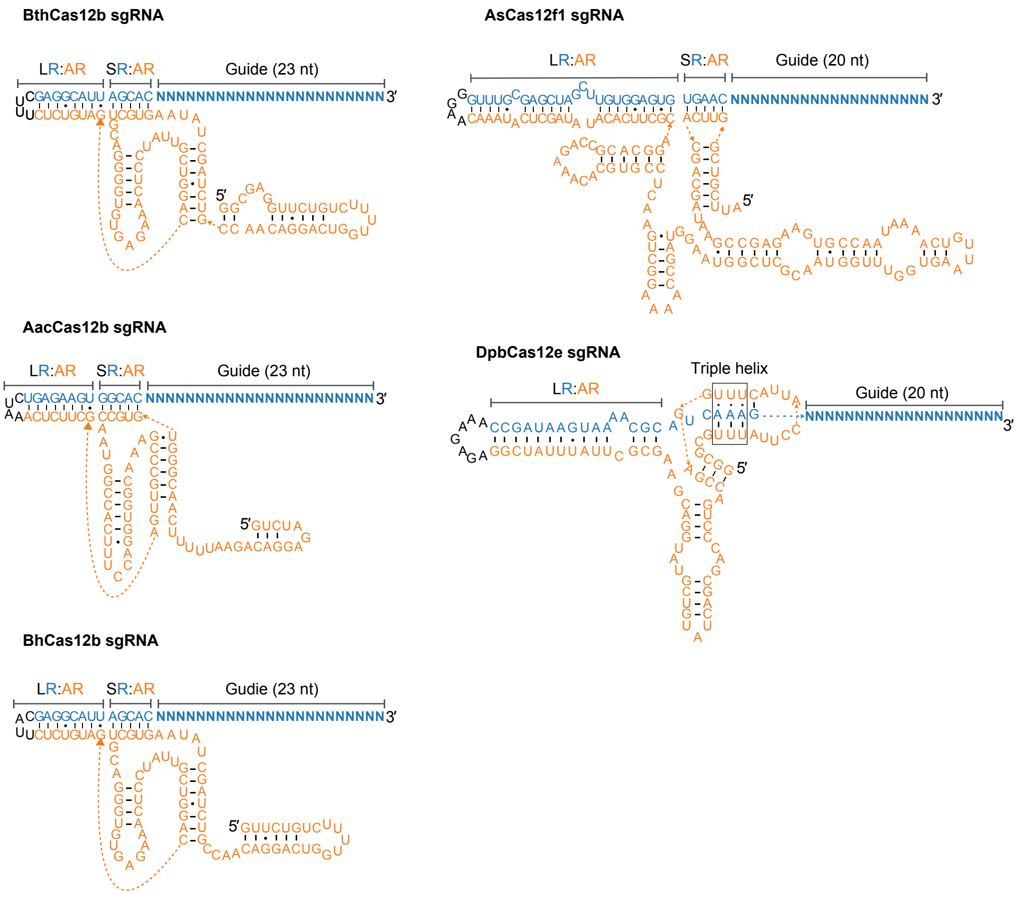

Supplementary Figure 1. The conformations of sgRNAs associated with Cas12 nucleases used in this work. The structures of the depicted sgRNAs are based on existing crystal structure information from *Bacillus thermoamylovorans* BthCas12b-sgRNA (PDB: 5WTI), *Alicyclobacillus acidoterrestris* AacCas12b-sgRNA (PDB: 5WQE), *Acidibacillus sulfuroxidans* AsCas12f1-sgRNA (PDB:8J12) and Deltaproteobacteria DpbCas12e-sgRNA (PDB: 6NY3). The structure of *Bacillus hisashii* BhCas12b-sgRNA is predicted based on structural information from its close relative, BthCas12b. SgRNAs are fused versions of tracrRNAs (in brown) and crRNAs (in blue). R:AR, repeat/anti-repeat.

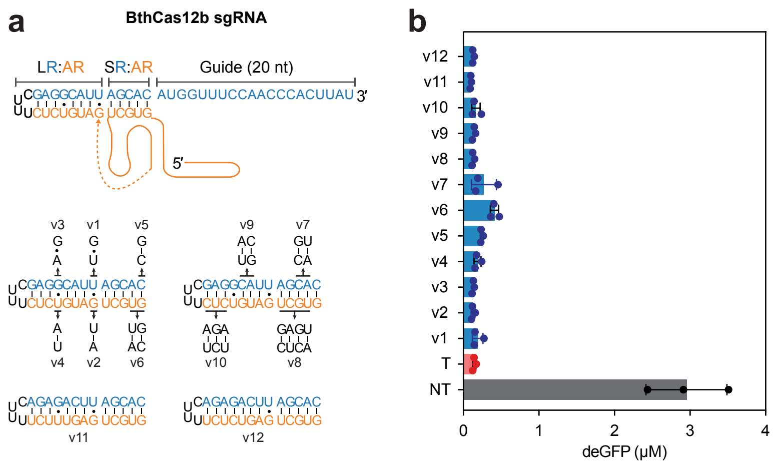

Supplementary Figure 2. The long and short RNA duplexes in the BthCas12b sgRNA can be reprogrammed. **a**, Depiction of BthCas12b sgRNAs with mutations that preserve the secondary structure. **b**, End-point measurements of GFP expression after a 16-hour TXTL reaction. The gRNA targets the sequence encoding the N501Y mutation in the SARS-CoV-2 N501Y variant. T, targeting guide; NT, non-targeting guide. v1-v12, sgRNA variants with partial or whole RNA duplexes changed. Bars and error bars represent the mean and standard deviation from three independently mixed TXTL reactions, with each dot representing one measurement. Source data are provided as a Source Data file.

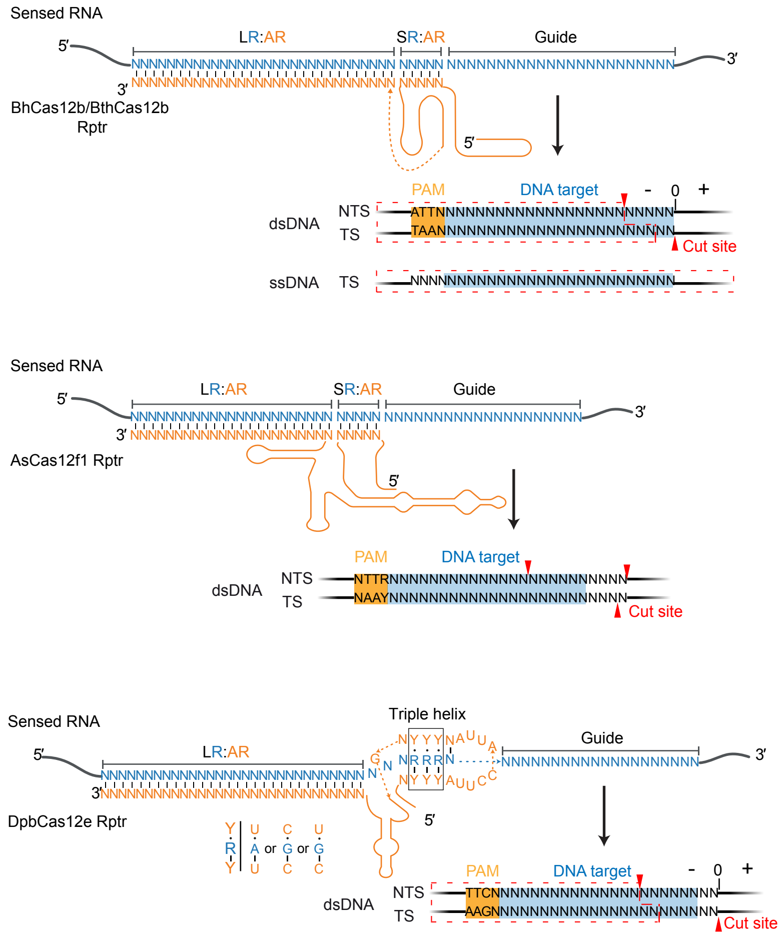

Supplementary Figure 3. Rptr design rules for BhCas12b, BthCas12b, AsCas12f1 and DpbCas12e. See the detailed description regarding Rptr design in the Methods sub-section “Design of Rptrs and dsDNA targets”. dsDNA target lengths of the target strand (TS) and non-target strand (NTS) are described using the cut site on the TS as a reference. The optimal DNA target sequences (60-nt ssDNA and 37-bp dsDNA NTS-6:TS-2 for BhCas12b, and NTS-8:TS-6 for DpbCas12e) identified in this work are boxed in red dashed lines.

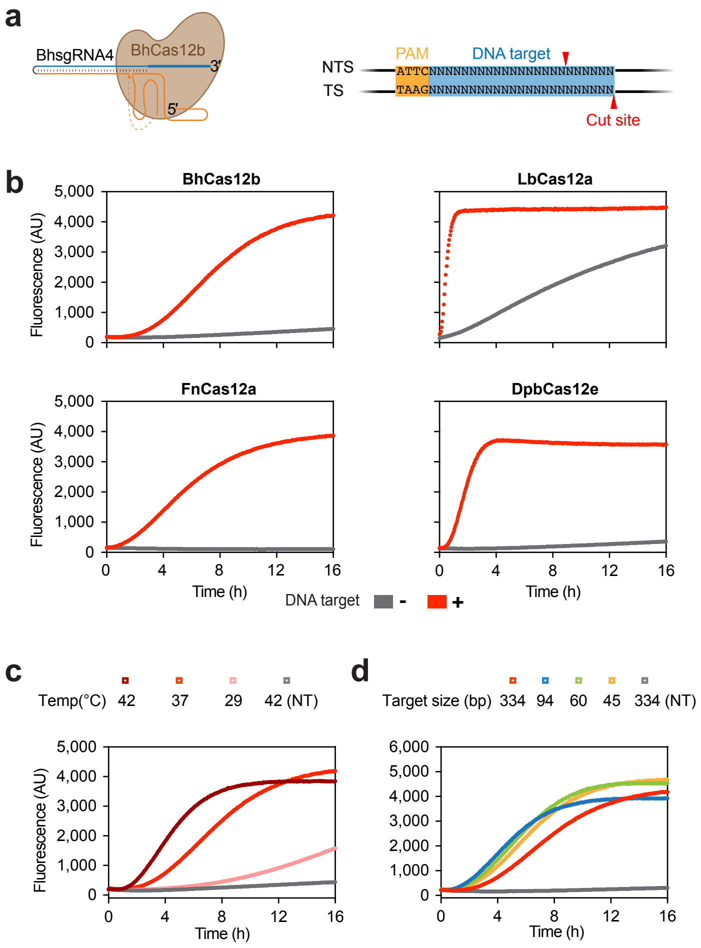

Supplementary Figure 4. Comparison of BhCas12b's collateral activity to other Cas12 orthologs and optimization of its activity by altering the reaction temperature and length of dsDNA targets. a, Depiction of the collateral cleavage assay. Purified BhCas12b, *in vitro* transcribed sgRNAs, linear dsDNA targets and fluorescent-labeled ssDNA reporters are used in this assay. See **Fig. 4a** for detailed information. **b**, Comparison of collateral cleavage activity among FnCas12a, BhCas12b, DpbCas12e, and LbCas12a using the same guide sequence in BhsgRNA4 for each nuclease based on *in vitro* collateral cleavage activity. **c**, Effect of temperature on *in vitro* collateral cleavage activity by BhCas12b. **d**, Impact of the length of target-encoding dsDNA on *in vitro* collateral cleavage activity by BhCas12b. NT, non-targeting sgRNA. The lengths for all DNA targets are based on the entire dsDNA, including the 27-bp spanning the PAM and DNA target sequence as well as the surrounding sequences. For the observed rate constant k_{obs} in **b**, **c** and **d, see Supplementary Data 1**. Curves in **b-d** represent the mean from two independent collateral assays. Source data are provided as a Source Data file.

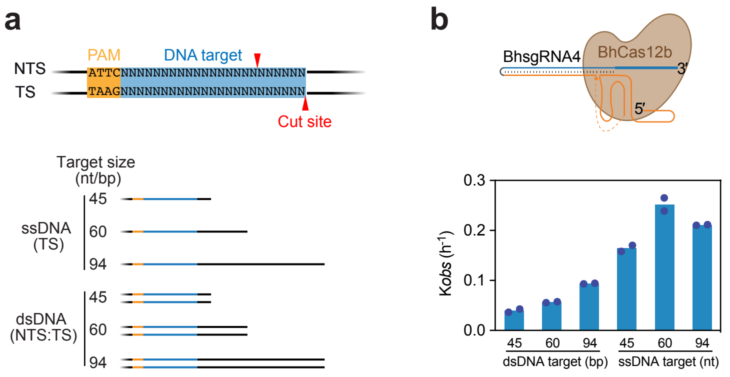

Supplementary Figure 5. BhCas12b exhibits enhanced collateral cleavage activity with ssDNA targets over dsDNA targets. **a**, Lengths for the tested ssDNA and dsDNA targets, including the 27 bp spanning the PAM and DNA target sequence as well as the surrounding sequences. ssDNA comprises only the TS. All DNA sequences are targets of BhsgRNA4. **b**, *In vitro* collateral cleavage activity of a fluorescent ssDNA molecular beacon with BhCas12b using the different ssDNA and dsDNA substrates. The collateral cleavage assay was conducted over 16 hours, which was used to calculate k_{obs} values. The k_{obs} values for each construct are shown in **Supplementary Data 1**. Dots represent individual measurements from two independent collateral cleavage assays. Bars represent the mean of the dots. Source data are provided as a Source Data file.

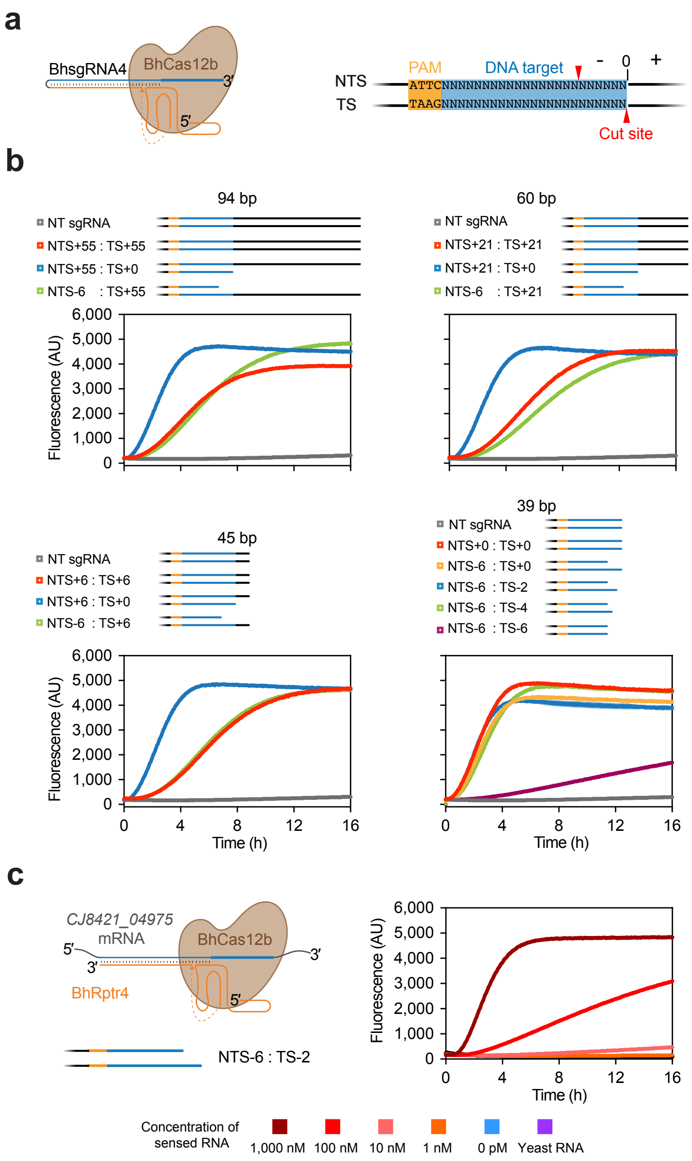

Supplementary Figure 6. Truncating the dsDNA target enhances collateral cleavage by BhCas12b. a, Depiction of the *in vitro* collateral cleavage assay using BhCas12b, BhsgRNA4, and different dsDNA target lengths and configurations. dsDNA target lengths of the target strand (TS) and non-target strand (NTS) are described using the cut site on the TS as a reference. **b**, Time-course measurements of *in vitro* collateral cleavage of a fluorescent ssDNA molecular beacon with BhCas12b using different dsDNA substrates. **c**, Detection of full-length *CJ8421_04975* mRNA using BhCas12b Rptr4 based on *in vitro* collateral cleavage activity. The data are plotted as 16-hour time-course curves. The plot in **Fig. 4c** is made using the end-point values at 16 hours. Yeast RNA is added in the same mass as the 1,000 nM sensed mRNA. For

the observed rate constant k_{obs} in **b** and **c,** see **Supplementary Data 1**. Curves in **b-c** represent the mean from two independent collateral assays. Source data are provided as a Source Data file.

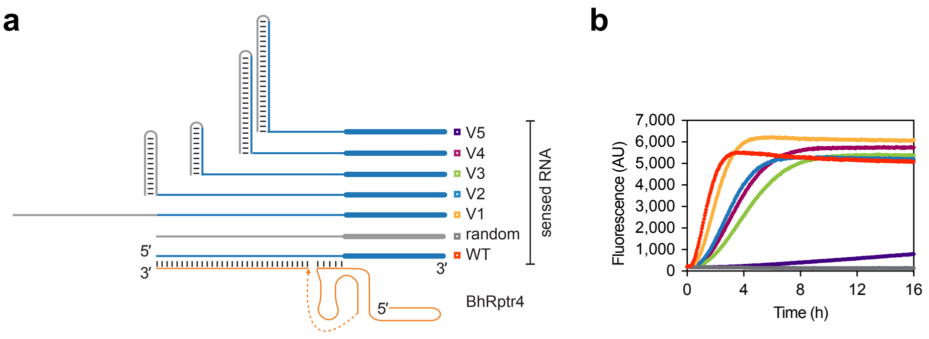

Supplementary Figure 7. Impact of secondary structure within the sensed RNA on collateral cleavage activity by BhCas12b. a, Depiction of sensed RNA with different sequences and predicted structure appended to the 5' end of the sensed RNA fragment from *CJ8421_04975* mRNA hybridizing with BhCas12b Rptr4. WT, no appended sequence; random, a random RNA sequence with the same length as the WT counterpart; V1-V5, modified sensed RNAs with varying-sized hairpins achieved by appending extra nucleotides or modifying nucleotides at the 5' end of the sensed RNA. **b**, Time-course measurements of *in vitro* collateral cleavage of a fluorescent ssDNA molecular beacon with BhCas12b. Colors match those shown on the left. Curves represent the mean from two independent collateral assays. Source data are provided as a Source Data file.

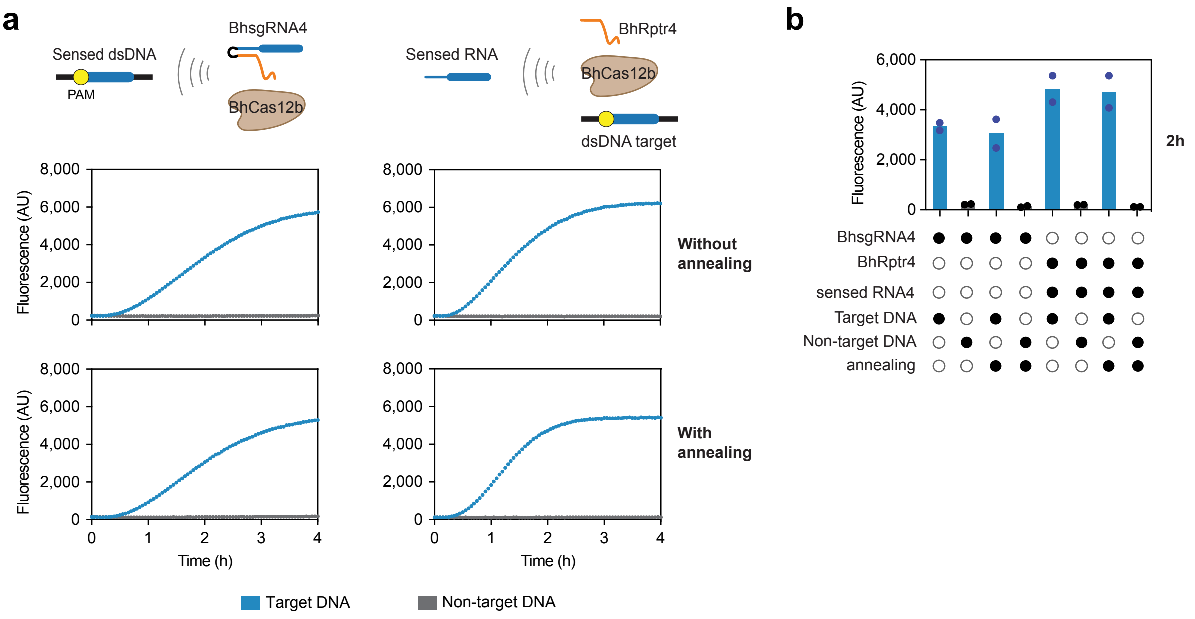

Supplementary Figure 8. Comparison of collateral cleavage activity with an sgRNA and sensed RNA-Rptr pair by BhCas12b with and without annealing. a. Time-course measurements of *in vitro* collateral cleavage of a fluorescent ssDNA molecular beacon with BhCas12b. BhCas12b was combined with either BhsgRNA4 or a Rptr-sensed RNA pair representing BhRptr4 and the corresponding sensed RNA fragment from *CJ8421_04975* mRNA. The sensed RNA and its associated Rptr were annealed at a 1:1 molar ratio in 1X NEBr2.1 buffer by heating at 95°C for 2 minutes and gradually cooling to 4°C over 30 minutes. The same annealing procedure was also applied to the sgRNA construct. **b.** Fluorescence values after 2 hours of reaction time. Solid circles, presence of components or procedures; Hollow circles, absence of components or procedures. Curves in **a** represent the mean from two independent collateral assays. Dots in **b** represent individual measurements from two independent collateral cleavage assays, while bars represent the mean of the dots. Source data are provided as a Source Data file.

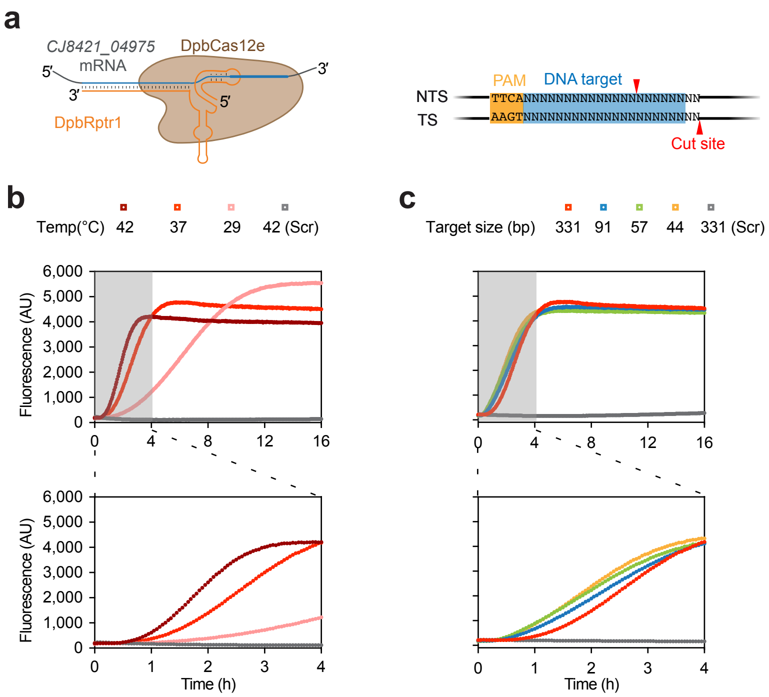

Supplementary Figure 9. Impact of temperature and DNA target shortening on collateral cleavage activity by DpbCas12e via altering reaction temperature and length of dsDNA targets. a, Depiction of the *in vitro* collateral cleavage assay using DpbCas12e, a sensed RNA-Rptr pair, and different dsDNA target configurations. Purified DpbCas12e, *in vitro* transcribed DpbRptr1 and *CJ8421_04975* mRNA, linear dsDNA targets and fluorescent-labeled ssDNA reporters are used in this assay. The lengths for all DNA targets are based on the entire DNA, including the 24-bp spanning the PAM and DNA target sequence as well as the surrounding sequences. **b**, Time-course measurements of *in vitro* collateral cleavage of a fluorescent ssDNA molecular beacon when varying the reaction temperature. The 331-bp DNA target was used. **c**, Time-course measurements of *in vitro* collateral cleavage of a fluorescent ssDNA molecular beacon when varying the DNA target length. All DNA targets have a blunt end and whose length is measured extending from the cut site on the non-target strand. Observed rate constant k_{obs} in **b** and **c** are reported in **Supplementary Data 1**. scr, Rptr with the RNA duplex and triple-helix sequence scrambled. Curves in **b-c** represent the mean from two independent collateral cleavage assays. Source data are provided as a Source Data file.

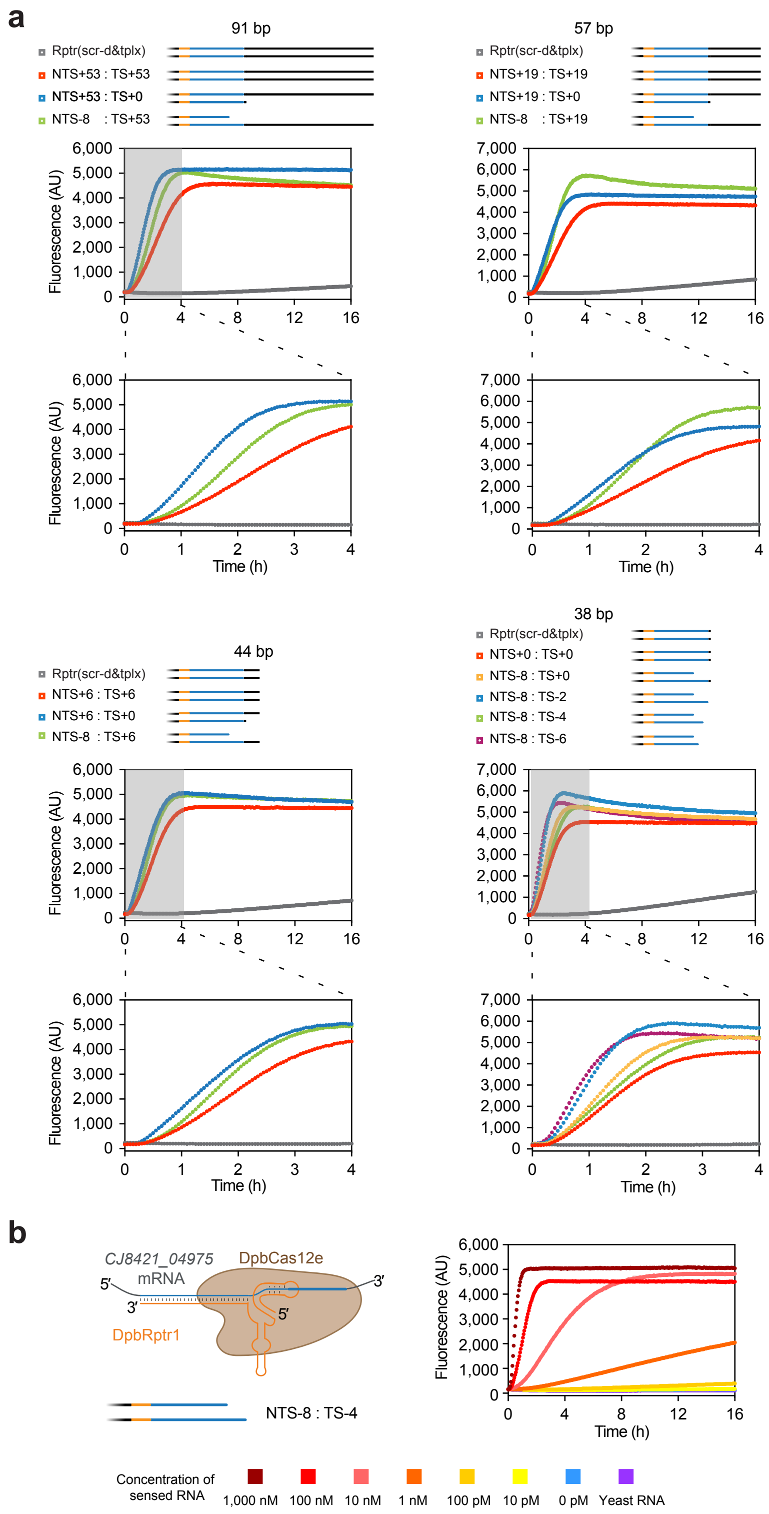

Supplementary Figure 10. Truncating the dsDNA target enhances collateral cleavage by DpbCas12e. a, Time-course measurements of *in vitro* collateral cleavage of a fluorescent ssDNA

molecular beacon when varying the DNA target lengths and configurations. The full-length *CJ8421_04975* mRNA-DpbRptr1 pair used was the same as those in Figures 4d and S10b. **b**, Time-course measurements for the detection of full-length *CJ8421_04975* mRNA using DpbCas12e Rptr1 based on *in vitro* collateral cleavage activity. The endpoints of the 16-hour time-courses are plotted in **Figure 4e**. Yeast RNA is added at the same mass as the 1,000 nM of sensed mRNA. Rptr(scr-d&tplx), Rptr with the RNA duplex and triple-helix sequence scrambled. The observed rate constant k_{obs} in **a**-**b** can be found in **Supplementary Data 1**. Curves represent the mean from two independent collateral assays. Source data are provided as a Source Data file.

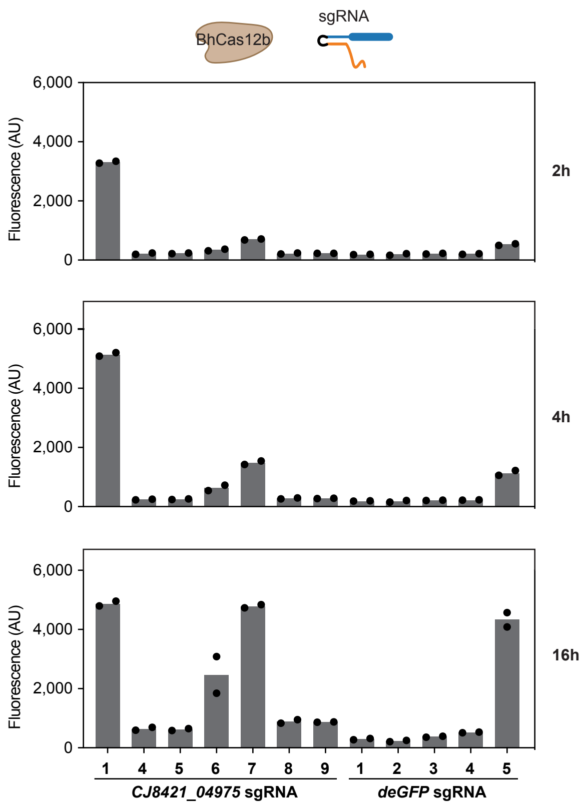

Supplementary Figure 11. BhCa12b sgRNAs exhibit ranging DNA target-independent collateral cleavage activities *in vitro*. All sgRNAs were designed to target the *CJ8421_04975* mRNA and the *deGFP* mRNA, even though no DNA target was included in the *in vitro* collateral cleavage assays. Two prior sgRNAs targeting the *CJ8421_04975* mRNA (BhsgRNA#1, 4 used in **Figure 5a**) serve as examples of high and low DNA target-independent collateral cleavage activity. Two new sgRNAs (*CJ8421_04975* sgRNA#7 and *deGFP*#5) showed background activity substantially higher than that of the low-background control *CJ8421_04975* sgRNA#4 at all three time points. Values at three time points (2h, 4h and 16h) were extracted from the 16-hour time-course to generate each plot. Curves represent the mean from two independent collateral assays. Source data are provided as a Source Data file.

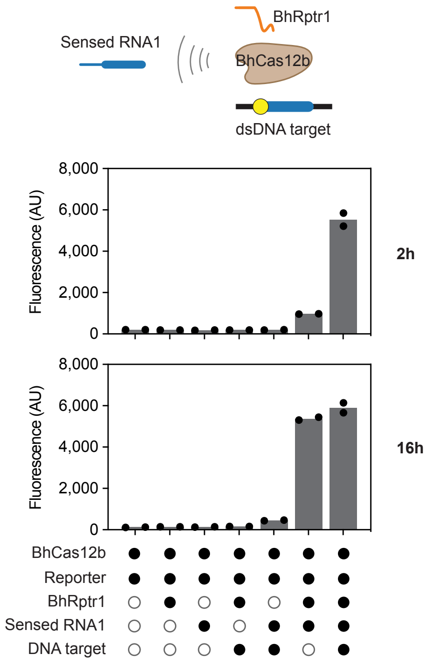

Supplementary Figure 12. Both the sensed RNA and Rptr are required to elicit DNA target-independent collateral cleavage activity *in vitro*. Values at two time points (2h and 16h) were extracted from the 16-hour time-course to generate the plots. Sensed RNA1 is a 59-nt sensed RNA fragment from the *CJ8421_04975* mRNA. Dots represent individual measurements from two independent collateral cleavage assays. Bars represent the mean of the dots. Source data are provided as a Source Data file.

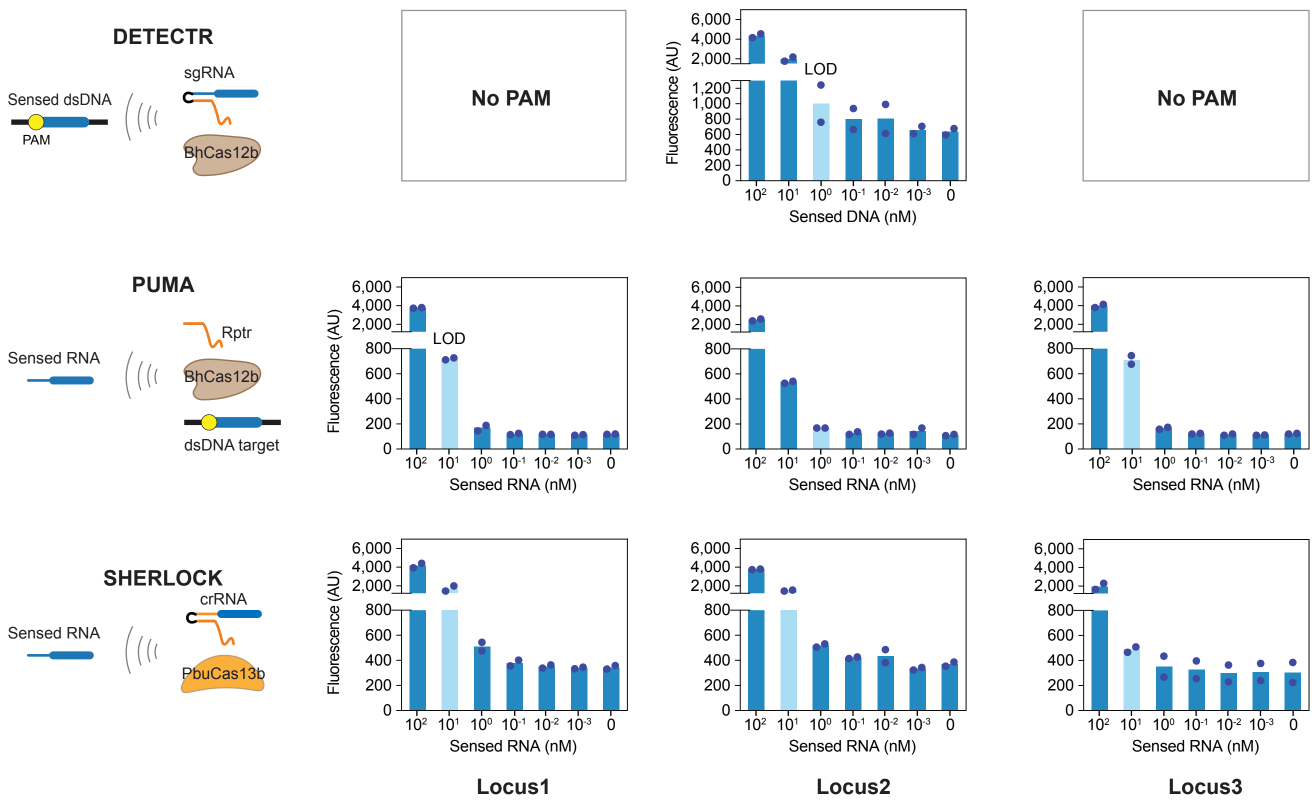

Supplementary Figure 13. PUMA exhibits detection sensitivity comparable to DETECTR and SHERLOCK in the absence of nucleic acid pre-amplification. BhCas12b was used for DETECTR to provide a direct comparison with PUMA. PbuCas13b was used for SHERLOCK. For DETECTR, locus 1 and locus 3 lack a consensus PAM sequence required by BhCas12b and thus were not tested. Dots represent individual endpoint measurements from two independent collateral cleavage assays conducted for 16 hours. Light blue bars indicate the limit-of-detection (LOD) conservatively estimated as the lowest concentration yielding an average fluorescence exceeding 50% of that of the no-RNA control. Source data are provided as a Source Data file.

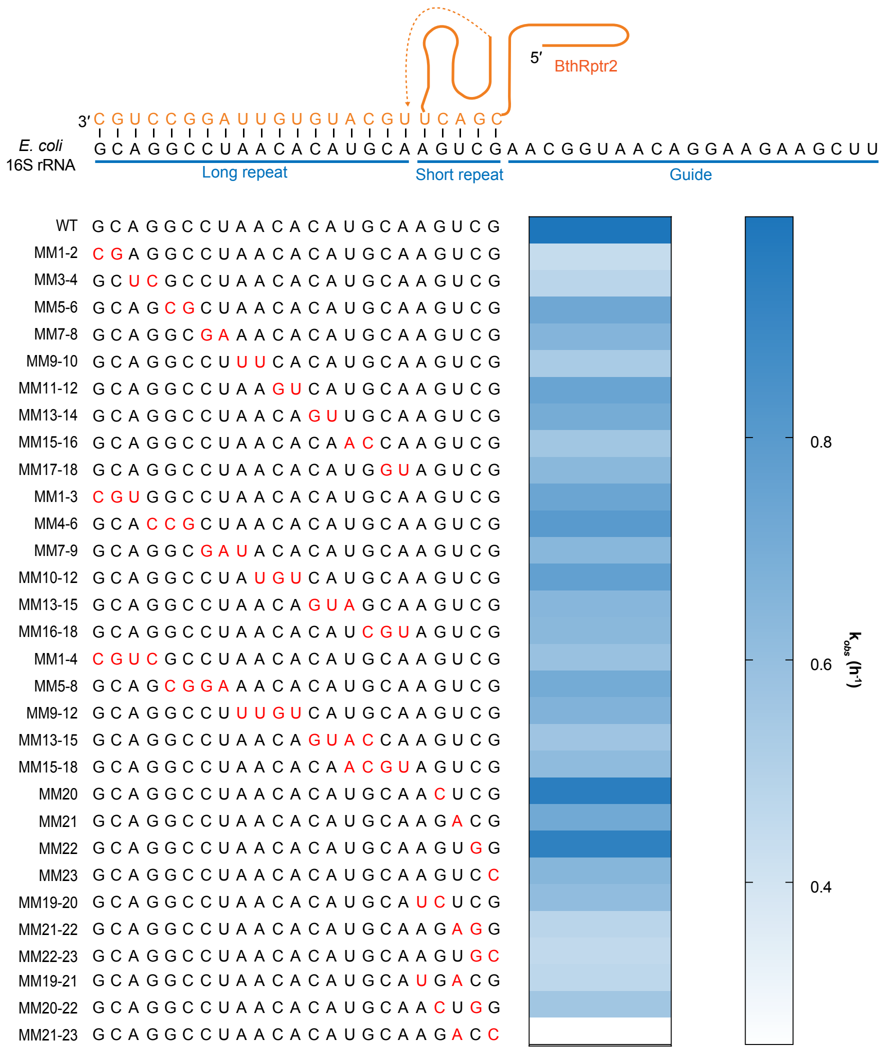

Supplementary Figure 14. BthCas12b tolerates mismatches in the repeat/anti-repeat duplex formed between a sensed RNA and Rptr. A fragment of *E. coli* 16S rRNA was used as the sensed RNA, while BthRptr2 was used as the Rptr. Two to four base pairs of continuous mismatches were introduced between the repeat and anti-repeat. Time-course measurements were collected as part of *in vitro* collateral cleavage of a fluorescent ssDNA molecular beacon with BthCas12b. Values represent the mean from two independent collateral cleavage assays. See the k_{obs} values in **Supplementary Data 1**. Source data are provided as a Source Data file.

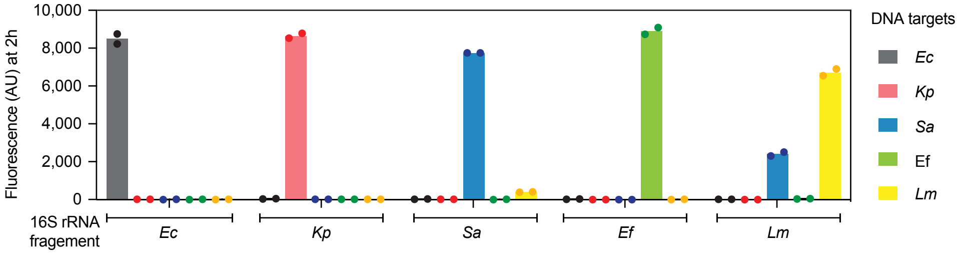

Supplementary Figure 15. Specific detection of pathogen 16S rRNAs with a universal Rptr and corresponding dsDNA targets. Time-course measurements were collected as part of *in vitro* collateral cleavage of a fluorescent ssDNA molecular beacon with BthCas12b. Values after 2 hours of reaction time are shown. Otherwise, conditions match those described in Figure 6c. Dots represent individual measurements from two independent collateral assays. Bars represent the mean of the dots. Source data are provided as a Source Data file.
