# Supplementary material for: TracrRNA reprogramming enables direct PAM-independent detection of RNA with diverse DNA-targeting Cas12 nucleases
Source: Nat Commun. 2024 Jul 13;15:5909. doi: 10.1038/s41467-024-50243-x (PMC11246509; doi:10.1038/s41467-024-50243-x)
Supplement: Supplementary file 3 — Description Of Additional Supplementary File [file 41467_2024_50243_MOESM3_ESM.pdf]

Description of Additional supplementary file

Supplementary Data 1. Values for k_{obs}.

Supplementary Data 2. List of strains, plasmids, gBlocks, amplified linear DNA, and oligonucleotides.
