# Supplementary material for: TracrRNA reprogramming enables direct PAM-independent detection of RNA with diverse DNA-targeting Cas12 nucleases
Source: Nat Commun. 2024 Jul 13;15:5909. doi: 10.1038/s41467-024-50243-x (PMC11246509; doi:10.1038/s41467-024-50243-x)
Supplement: Supplementary file 6 — Reporting Summary [file 41467_2024_50243_MOESM6_ESM.pdf]

nature portfolio

Corresponding author(s): Chase L. Beisel
Last updated by author(s): Jul 4, 2024

Reporting Summary

Nature Portfolio wishes to improve the reproducibility of the work that we publish. This form provides structure for consistency and transparency in reporting. For further information on Nature Portfolio policies, see our Editorial Policies and the Editorial Policy Checklist.

Statistics

For all statistical analyses, confirm that the following items are present in the figure legend, table legend, main text, or Methods section.

n/a	Confirmed	
☐	☒	The exact sample size (*n*) for each experimental group/condition, given as a discrete number and unit of measurement
☐	☒	A statement on whether measurements were taken from distinct samples or whether the same sample was measured repeatedly
☐	☒	The statistical test(s) used AND whether they are one- or two-sided<br>*Only common tests should be described solely by name; describe more complex techniques in the Methods section.*
☒	☐	A description of all covariates tested
☐	☒	A description of any assumptions or corrections, such as tests of normality and adjustment for multiple comparisons
☐	☒	A full description of the statistical parameters including central tendency (e.g. means) or other basic estimates (e.g. regression coefficient) AND variation (e.g. standard deviation) or associated estimates of uncertainty (e.g. confidence intervals)
☐	☒	For null hypothesis testing, the test statistic (e.g. *F*, *t*, *r*) with confidence intervals, effect sizes, degrees of freedom and *P* value noted<br>*Give P values as exact values whenever suitable.*
☒	☐	For Bayesian analysis, information on the choice of priors and Markov chain Monte Carlo settings
☒	☐	For hierarchical and complex designs, identification of the appropriate level for tests and full reporting of outcomes
☒	☐	Estimates of effect sizes (e.g. Cohen's *d*, Pearson's *r*), indicating how they were calculated

Our web collection on statistics for biologists contains articles on many of the points above.

Software and code

Policy information about availability of computer code

Data collection	No software or code was used for data collection.
Data analysis	Prism 9 (Graphpad) was used to extrapolate kobs values. No other software or code was used for data analysis.

For manuscripts utilizing custom algorithms or software that are central to the research but not yet described in published literature, software must be made available to editors and reviewers. We strongly encourage code deposition in a community repository (e.g. GitHub). See the Nature Portfolio guidelines for submitting code & software for further information.

Data

Policy information about availability of data

All manuscripts must include a data availability statement. This statement should provide the following information, where applicable:

- Accession codes, unique identifiers, or web links for publicly available datasets
- A description of any restrictions on data availability
- For clinical datasets or third party data, please ensure that the statement adheres to our policy

Source data are included in this paper. Crystal structures used in this work include PDB 7VYX [https://doi.org/10.2210/pdb7VYX/pdb], 5WTI [https://doi.org/10.2210/pdb5WTI/pdb], 5WQE [https://doi.org/10.2210/pdb5WQE/pdb], 8J12 [https://doi.org/10.2210/pdb8J12/pdb] and 6NY3 [https://doi.org/10.2210/pdb6NY3/pdb].

Research involving human participants, their data, or biological material

Policy information about studies with human participants or human data. See also policy information about sex, gender (identity/presentation), and sexual orientation and race, ethnicity and racism.

Reporting on sex and gender	No human participants, data or biological material
Reporting on race, ethnicity, or other socially relevant groupings	No human participants, data or biological material
Population characteristics	No human participants, data or biological material
Recruitment	No human participants, data or biological material
Ethics oversight	No human participants, data or biological material

Note that full information on the approval of the study protocol must also be provided in the manuscript.

Field-specific reporting

Please select the one below that is the best fit for your research. If you are not sure, read the appropriate sections before making your selection.

☒ Life sciences ☐ Behavioural & social sciences ☐ Ecological, evolutionary & environmental sciences

For a reference copy of the document with all sections, see nature.com/documents/nr-reporting-summary-flat.pdf

Life sciences study design

All studies must disclose on these points even when the disclosure is negative.

Sample size	No statistical methods were used to predetermine sample size. At least two independent replicates were performed for in vitro collateral cleavage assays, and three independently mixed replicates were performed for TXTL experiments. Sample sizes were determined based on our previous experiences and what were described in similar experiments in published papers.
Data exclusions	No data were excluded from the analyses with the exception of individual TXTL runs yielding no fluorescence above background; these instances are marked with a dash in the Source Data.
Replication	Values in figures represent individual measurements from three independently mixed TXTL reactions or from two independent collateral assays.
Randomization	Randomization was not applied in line with standard experimental procedures in molecular biology.
Blinding	Blinding was not applied in line with standard experimental procedures in molecular biology.

Reporting for specific materials, systems and methods

We require information from authors about some types of materials, experimental systems and methods used in many studies. Here, indicate whether each material, system or method listed is relevant to your study. If you are not sure if a list item applies to your research, read the appropriate section before selecting a response.

Materials & experimental systems

n/a	Involved in the study	
☒	☐	Antibodies
☒	☐	Eukaryotic cell lines
☒	☐	Palaeontology and archaeology
☒	☐	Animals and other organisms
☒	☐	Clinical data
☒	☐	Dual use research of concern
☒	☐	Plants

Methods

n/a	Involved in the study	
☒	☐	ChIP-seq
☒	☐	Flow cytometry
☒	☐	MRI-based neuroimaging

Plants

Seed stocks	No plants were used this study.
Novel plant genotypes	No plants were used this study.
Authentication	No plants were used this study.
